# Selective Killing of Tumors Deficient in Methylthioadenosine Phosphorylase: A Novel Strategy

**DOI:** 10.1371/journal.pone.0005735

**Published:** 2009-05-29

**Authors:** Martin Lubin, Adam Lubin

**Affiliations:** 1 Dartmouth Medical School, Hanover, New Hampshire, United States of America; 2 Amtek, Hanover, New Hampshire, United States of America; Karolinska Institutet, Sweden

## Abstract

**Background:**

The gene for methylthioadenosine phosphorylase (MTAP) lies on 9p21, close to the gene CDKN2A that encodes the tumor suppressor proteins p16 and p14ARF. MTAP and CDKN2A are homozygously co-deleted, with a frequency of 35 to 70%, in lung and pancreatic cancer, glioblastoma, osteosarcoma, soft-tissue sarcoma, mesothelioma, and T-cell acute lymphoblastic leukemia. In normal cells, but not in tumor cells lacking MTAP, MTAP cleaves the natural substrate, 5′-deoxy-5′-methylthioadenosine (MTA), to adenine and 5-methylthioribose-1-phosphate (MTR-1-P), which are then converted to adenine nucleotides and methionine. This distinct difference between normal MTAP-positive cells and tumor MTAP-negative cells led to several proposals for therapy. We offer a novel strategy in which both MTA and a toxic adenine analog, such as 2,6-diaminopurine (DAP), 6-methylpurine (MeP), or 2-fluoroadenine (F-Ade), are administered. In MTAP-positive cells, abundant adenine, generated from supplied MTA, competitively blocks the conversion of an analog, by adenine phosphoribosyltransferase (APRT), to its active nucleotide form. In MTAP-negative tumor cells, the supplied MTA cannot generate adenine; hence conversion of the analog is not blocked.

**Principal Findings:**

We show that this combination treatment – adenine analog plus MTA – kills MTAP-negative A549 lung tumor cells, while MTAP-positive human fibroblasts (HF) are protected. In co-cultures of the breast tumor cell line, MCF-7, and HF cells, MCF-7 is inhibited or killed, while HF cells proliferate robustly. 5-fluorouracil (5-FU) and 6-thioguanine (6-TG) may also be used with our strategy. Though neither analog is activated by APRT, in MTAP-positive cells, adenine produced from supplied MTA blocks conversion of 5-FU and 6-TG to their toxic nucleotide forms by competing for 5-phosphoribosyl-1-pyrophosphate (PRPP). The combination of MTA with 5-FU or 6-TG, in the treatment of MTAP-negative tumors, may produce a significantly improved therapeutic index.

**Conclusion:**

We describe a selective strategy to kill tumor cells lacking MTAP.

## Introduction

**Figure 1:**
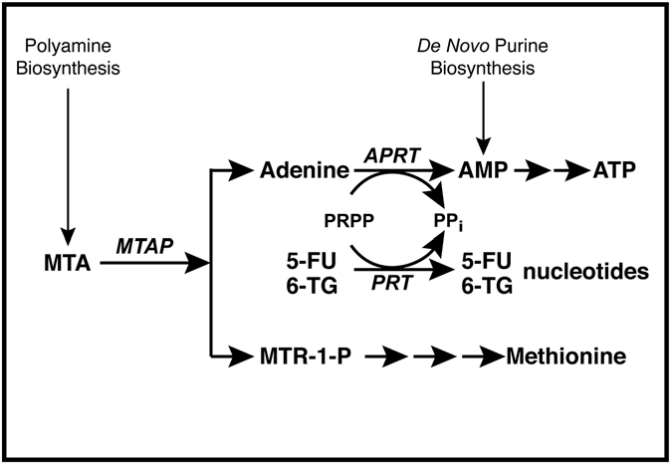
MTAP metabolic pathway. In normal cells, MTAP cleaves MTA, a by-product of polyamine biosynthesis, into adenine and MTR-1-P. Adenine is converted to AMP by the ubiquitous enzyme APRT, with PRPP serving as donor of the phosphoribosyl group. MTR-1-P is converted by a series of steps to methionine. AMP is also produced in cells by *de novo* purine biosynthesis. In addition to APRT, other cellular phosphoribosyltransferases (PRTs), such as hypoxanthine-guanine phsophoribosyl transferase and orotate phosphoribosyltranferase, covert purines and pyrimidines to nucleotides.

MTA was discovered almost one hundred years ago, and its correct structure described not long after. But fifty years passed before Pegg and Williams-Ashman found the enzyme MTAP that catalyzes the phosphorolysis of MTA to adenine and MTR-1-P [1]. This pathway is the only known source of free cellular adenine. MTA is produced from S-adenosyl-L-methionine during synthesis of the polyamines, spermidine and spermine. Both products of MTA cleavage by MTAP are reused: adenine is returned to the adenine nucleotide pool through the enzyme APRT, and MTR-1-P is metabolized, by a series of additional steps, to methionine and formate (Figure 1). Thus MTAP plays a crucial role in recycling of the adenine and methylthio moieties of MTA back to the metabolites from which S-adenosyl-L-methionine is formed – ATP and methionine. All normal mammalian tissues contain MTAP – at least no exceptions have been found – and this enzyme keeps cellular MTA at very low levels [2].

After Toohey found that some murine leukemia cell lines lack MTAP [3], others reported that many of the most common human tumors are also deficient in MTAP. This deficiency occurs because the MTAP gene is often homozygously co-deleted with the CDKN2A gene, which encodes the tumor suppressor proteins p16 and p14ARF. The common occurrence of MTAP-deficiency in some of those tumors that have been most resistant to treatment is striking. About 40% of non-small cell lung, pancreatic, and biliary tract cancer, 70% of mesothelioma and glioblastoma, and 35% of osteosarcoma, soft-tissue sarcoma, and T-cell acute lymphoblastic leukemia lack MTAP [4]–[11].

In malignant melanoma, loss of MTAP-expression is also common – with one report placing it as high as 60% – but in this tumor the deficiency appears to be due to promoter hypermethylation [12]. Information on the incidence of MTAP-deficiency in breast cancer is still limited, but of nine cell lines tested, four, including the often-studied MCF-7 and MDA-MB-231, were MTAP-negative [13].

The discovery that MTAP is often absent in tumors led to proposals for a selective therapy that would inhibit or kill tumor cells, but leave host cells relatively unharmed. Two distinct approaches were suggested. In the first, Carson and colleagues showed that inhibitors of *de novo* purine synthesis, methotrexate or azaserine, in combination with MTA, selectively killed MTAP-negative cells, while sparing MTAP-positive cells which could derive their purine nucleotide requirements from the supplied MTA [14]. A number of studies that followed up on this proposal examined other inhibitors of *de novo* purine synthesis, such as 5,10-dideazotetrahydrofolate and L-alanosine, an inhibitor of AMP synthesis from IMP [15].

To determine whether inhibitors of purine biosynthesis might act selectively against MTAP-negative tumors, two multi-center Phase I/II clinical trials of L-alanosine were started, but both trials were suspended early [16], [17]. A recent report on one of these abbreviated trials concluded that this treatment was ineffective [18].

In the second approach, Tisdale proposed that if serum were depleted of methionine by a methioninase, normal cells, but not MTAP-negative tumor cells, could be rescued by providing MTA, which is cleaved to MTR-1-P and leads to methionine synthesis [19]. As yet, there have been no reported clinical trials of this proposed therapy.

We have developed a novel strategy that takes advantage of MTA metabolism to provide a selective therapy of MTAP-negative tumors (Fig. 1). Our strategy involves two agents: a purine or pyrimidine analog toxic to *both* MTAP-negative tumor cells and MTAP-positive normal cells, and a second agent that protects the normal cells.

The adenine analogs DAP, MeP, and F-Ade are especially effective with our method. In normal, non-tumor cells, when MTA, or another MTAP substrate, 5′-deoxyadenosine (5′-dAdo), is present in sufficient amount, the abundant adenine produced from these substrates, by the action of MTAP, competes with these analogs for phosphoribosylation by APRT and the cells are protected from toxicity. In MTAP-negative tumor cells, adenine is not produced from MTA or 5′-dAdo, and the cells are killed by the adenine analog.

DAP was actually the first purine analog synthesized by Elion and Hitchings and put into clinical trials at Memorial Sloan-Kettering in the late 1940s, where it produced two good clinical remissions in chronic granulocytic leukemia and one remarkable long-term remission in a patient with acute leukemia, after only three weeks of drug treatment [20]. Because two other patients showed toxic side effects, however, clinical application of DAP was abandoned.

MeP and F-Ade have also been of recent interest because of their marked potency. Even single injections of MeP into tumors growing in immunodeficient mice have shown pronounced and prolonged antitumor effects [21]. Both analogs have also been prepared as riboside prodrugs. When isolated pancreatic or glioma tumors were transduced with a genetically engineered purine nucleoside phosphorylase, MeP or F-Ade was released from the prodrugs, producing a marked reduction in tumor size [22].

Other purine and pyrimidine analogs also require PRPP for conversion, by cellular phosphoribosyltransferases (PRTs), to their toxic nucleotides. In MTAP-positive, but not MTAP-negative cells, adenine derived from MTA (or another MTAP substrate) competes with such analogs for reaction with PRPP in their respective phosphoribosylations, thus blocking the conversion of these drugs to their nucleotides.

Two clinical cancer drugs – 5-FU and 6-TG – might thus be used in our proposed strategy. In the case of 6-TG, the PRT is hypoxanthine-guanine phosphoribosyltransferase. For 5-FU, several different pathways can be involved in activation but a principal one is conversion by orotate phosphoribosyltransferase to the nucleotide of 5-FU. If MTA or 5′-dAdo is supplied to an MTAP-positive cell, the increased flux of adenine, through the APRT-pathway, would reduce available cellular PRPP and thus limit the conversion of 5-FU and 6-TG to their toxic nucleotides. Consistent with this idea, previous *in vitro* studies did indeed find that adenine reduces the toxicity of 5-FU and 6-TG [23], [24] and one report describes the same effect in mice [25]. 

Our proposed strategy may allow the use of high drug concentrations, lethal to MTAP-negative tumors, but with decreased toxicity to host cells.

## Results

### Selective killing of MTAP-negative cells by adenine analogs

**Figure 2:**
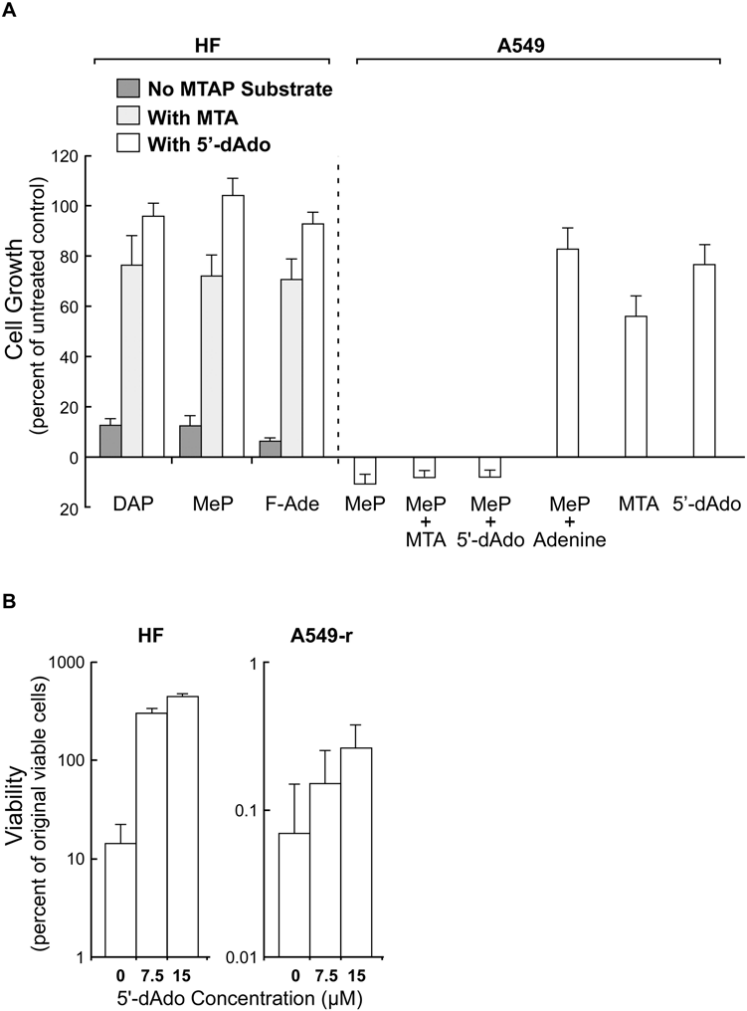
MTA and 5′-dAdo protect MTAP-positive HF, but not MTAP-negative A549 cells, from toxic adenine analogs. (A) HF cells were cultured with the adenine analogs DAP (100 µM), MeP (5 µM), and F-Ade (0.3 µM) with either MTA (15 µM) or 5′-dAdo (15 µM) for three days. Dishes were then trypsinized and cell numbers measured with a Coulter counter. Data from three independent experiments, bars SE. MTAP-negative A549 cells were cultured for three days with MeP (5 µM) and either MTA (15 µM), 5′-dAdo (15 µM), or adenine (15 µM). As controls, cells were cultured with MTA alone (15 µM) or 5′-dAdo alone (15 µM). Dishes were then trypsinized and cell numbers measured with a Coulter counter. Data from three independent experiments, bars SE. (B) For co-culture experiments, the ouabain-resistant cell line A549-r was used. Stocks of HF and A549-r cells were trypsinized and cell numbers counted to determine the amount of each cell line to be seeded for co-culture. To measure the initial number of viable cells, each cell line was separately seeded, in duplicate, into dishes for clonogenic assay. For co-cultures, each dish had 45,000 A549-r and 3,000 HF cells, with DAP at 125 µM, and 5′-dAdo at 0, 7.5, or 15 µM. After three days of incubation, two dishes were trypsinized and serially diluted into fresh medium, with adenine (15 µM) added to suppress further action of DAP, for clonogenic assay of viable A549-r clones. After two additional days of culture, ouabain (0.1 µM) was added to these dishes to inhibit or kill HF cells, while permitting the small number of surviving A540-r cells to grow and form large clones. The remaining two dishes did not receive ouabain and hence viable HF clones could grow and be identified by staining. Counts from duplicate dishes were averaged. Data from two independent experiments, bars SE.

MTAP-positive HF cells were cultured for three days with DAP (100 µM), MeP (5 µM), or F-Ade (0.3 µM), with or without the MTAP substrates MTA or 5′-dAdo. The adenine analogs produced strong inhibition of growth of HF cells. This inhibition was almost entirely prevented by 15 µM MTA or 5′-dAdo (Figure 2A). Adenine at 15 µM gave results similar to those with 5′-dAdo (data not shown).

MTA alone caused modest inhibition of HF cell growth. After 3 days at 15 µM, growth reached 85% of control; at 30 µM, 75%. In contrast, 5′-Ado, tested at concentrations as high as 150 µM, did not significantly inhibit growth.

Unlike MTAP-positive HF cells, MTAP-negative A549 cells, treated with the adenine analog MeP (5 µM), were not protected by the addition of MTA or 5′-dAdo (Figure 2A). Three controls – MeP (5 µM) with adenine (15 µM), MTA alone (15 µM), and 5′-dAdo alone (15 µM) – as expected, showed growth nearly equal to that of untreated cells. Similar results were found with the analogs DAP and F-Ade (data not shown).

### Selective killing of MTAP-negative cells in co-culture with MTAP-positive cells

To quantitatively assess selective killing of tumor cells when they are present in excess of normal cells, co-culture experiments were designed. For this purpose, a ouabain-resistant cell line, A549-r, was used to facilitate determination of viable tumor clones in the presence of HF cells.

45,000 A549-r and 3,000 HF cells were cultured, in quadruplicate, with DAP at 125 µM, and 5′-dAdo at 0, 7.5, or 15 µM. After three days of incubation, two of the four dishes were trypsinized and serially diluted into fresh medium, with added adenine (15 µM), for clonogenic assay of viable A549-r cells. After two additional days of culture, ouabain (0.1 µM) was added to inhibit or kill HF cells; without ouabain it would have been difficult to count a small number of surviving A549-r clones among a large number of growing HF cells. After several weeks in culture, A549-r clones were sufficiently large to be seen readily after staining with crystal violet. The remaining two dishes were serially diluted into medium, with adenine (15 µM), but no ouabain was added. Hence viable HF clones could grow and be counted.

Treatment of HF cell cultures with DAP for three days decreased the number of colony–forming cells to about 15% of the initial value. But with 5′-dAdo (7.5 or 15 µM) added to DAP, HF viability increased about three-fold, showing protection of HF cells from toxicity of DAP. In contrast, when 5′-dAdo and DAP were added to cultures of MTAP-negative A549-r cultures, viability of A549-r cells decreased markedly, and dishes that initially contained 45,000 viable cells showed less than 200 clones (Figure 2B).

**Figure 3:**
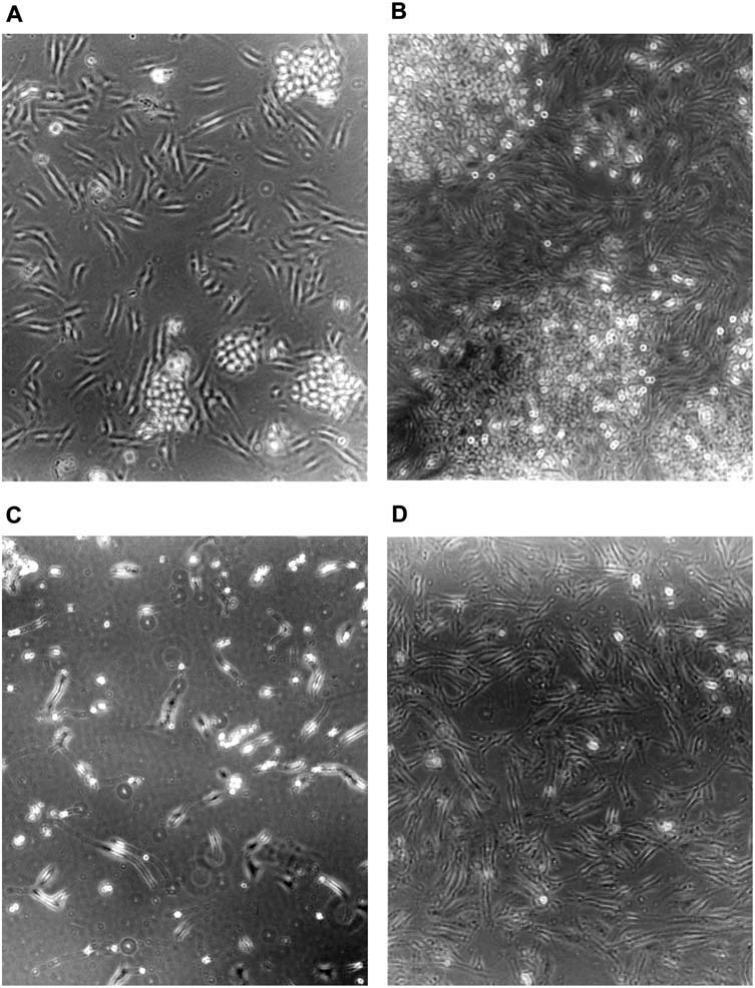
Strategy applied to co-culture of MTAP-negative MCF-7 and MTAP-positive HF cells. MCF-7 and HF cells were co-cultured with drugs added as follows: (A) Start of co-culture. (B) Three days without drugs. (C) Three days with DAP (200 µM). (D) Three days with DAP (200 µM) and MTA (50 µM). Representative fields are shown.

**Figure 4:**
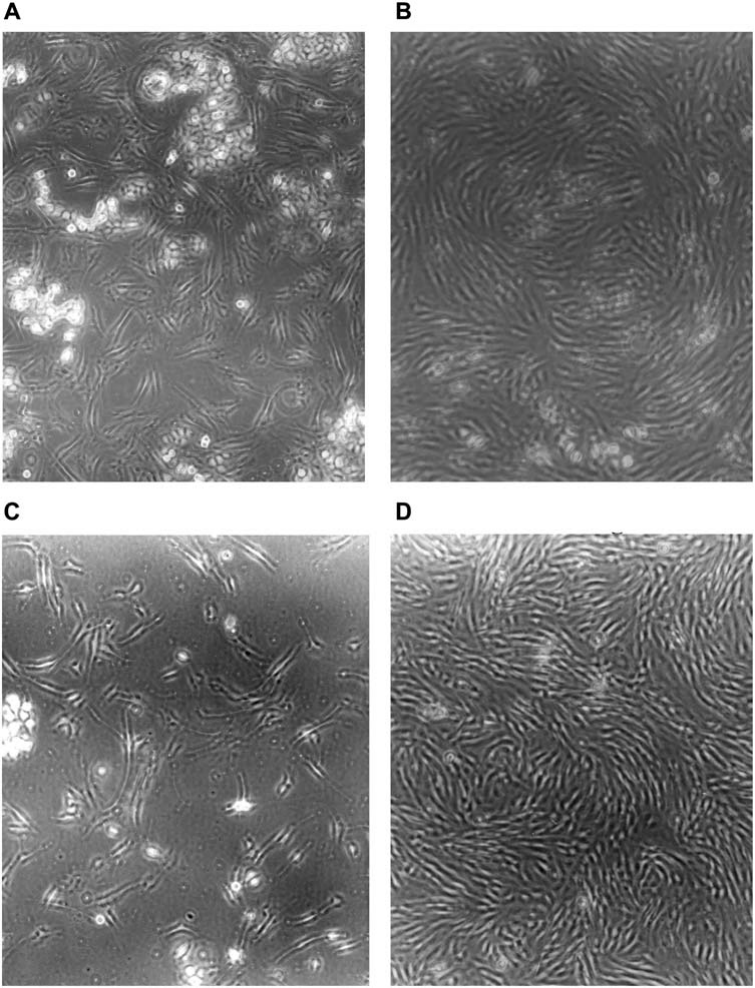
Strategy with prolonged subculture applied to co-culture of MTAP-negative MCF-7 and MTAP-positive HF cells. MCF-7 and HF cells were co-cultured with drugs added as follows: (A) Start of co-culture. (B) Four days with DAP (100 µM) and MTA (25 µM). (C) Co-culture in panel B split 1:5 into drug-free medium and fixed two days later. (D) Same as in panel C except culture fixed after five days. Representative fields are shown.

To determine whether selective killing of tumor cells could be demonstrated with a different MTAP-negative tumor cell line, we co-cultured HF and MCF-7 for three days. With no additions, both the fiber-like HF and the larger clumps of tumor cells grew robustly (Figures 3A, 3B). With just DAP alone, both HF and MCF-7 appeared to be dead, and subcultures confirmed that they were (Figure 3C and data not shown). But in the presence of protective MTA, while MCF-7 cells were killed, HF cells continued to proliferate (Figure 3D). In a similar experiment, HF and MCF-7, in the presence of DAP and MTA, were co-cultured for four days and then subcultured for an additional five days, without drugs, to assess survival of MCF-7 (Figure 4A,B,C,D). By then, only HF cells could be seen, without any evidence of MCF-7 clones (Figure 4D).

The results of the co-culture experiments with HF and MTAP-negative tumor cells, in the presence of the toxic analog DAP and the protective agents 5′-dAdo or MTA, are significant (Figures 2B, 3, and 4). They suggest that if this strategy is to be applied *in vivo*, the presence of large numbers of normal host cells will not interfere substantially with selective killing of tumor cells.

### Other analogs that compete for PRPP

**Figure 5:**
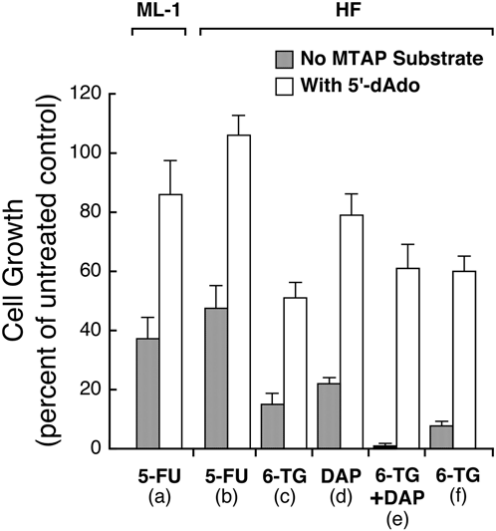
Protection by 5′-dAdo from toxicity of 5-FU and 6-TG in MTAP-positive ML-1 and HF cells. (a) ML-1 cells were cultured in RPMI 1640 with 10% FBS, for three days, with 5-FU (3 µM), with or without 5′-dAdo (60 µM). Cell numbers were then measured with a Coulter counter. Data from three independent experiments, bars SE. (b) HF cells were cultured in DMEM with 10% FBS for four days, with 5-FU (3 µM), with or without 5′-dAdo (60 µM). Dishes were then trypsinized and cell numbers measured. Data from three independent experiments, bars SE. (c, d, e) HF cells were cultured in DMEM with 10% FBS for three days with either 6-TG (20 µM), DAP (50 µM), or both, with or without 5′-dAdo (15 µM). Dishes were then trypsinized and cell numbers measured with a Coulter counter. Data from three independent experiments, bars SE. (f) HF cells were cultured in DMEM with 10% FBS for three days with 6-TG (40 µM), with or without 5′-dAdo (15 µM). Data from three independent experiments, bars SE.

As outlined in the introduction, MTAP substrates were expected to protect MTAP-positive cells from toxicity of the analogs 5-FU or 6-TG. Adenine produced from these substrates would compete with 5-FU or 6-TG for PRPP, and prevent conversion of the analogs to their nucleotides. To test this prediction, we treated MTAP-positive cells ML-1 and HF cells with 5-FU (3 µM). This concentration of 5-FU inhibited ML-1 and HF growth by about 50%. The addition of 5′-dAdo (60 µM) resulted in a doubling of growth, to levels about equal to that in untreated cultures (Figure 5a,b). At a higher concentration of 5-FU (10 µM) we could not demonstrate protection from toxicity by 5′-dAdo, and at a lower concentration of 5-FU (1 µM), cell growth was not inhibited (data not shown).

We also tested 6-TG (20 µM) and found that 5′-dAdo provided strong protection of HF cells (Figure 5c). With DAP at 50 µM, a concentration that does not cause maximum inhibition of growth, 5′-dAdo, as expected, protected HF cells from toxicity (Figure 5d). The combination of 6-TG and DAP at these concentrations was strongly inhibitory, but 5′-dAdo still provided strong protection from toxicity (Figure 5e). This result shows that drugs with different metabolic routes may act synergistically against MTAP-negative tumors and that MTAP-positive cells can be protected from these combinations. At a higher concentration of 6-TG (40 µM), 5′-dAdo (15 µM) still protected HF cells. Last, the experiments with 6-TG were repeated with MTA as the MTAP substrate, with results similar to those found with 5′-dAdo (data not shown).

## Discussion

### Summary of our findings

We demonstrate the salient features of a proposed strategy for treating tumors deficient in MTAP. The MTAP substrates, MTA and 5′-dAdo, protect MTAP-positive HF cells from toxicity of the adenine analogs DAP, MeP, and F-Ade (Figure 2A). In similar experiments with MTAP-negative A549 cells, no such protection occurred (Figure 2A).

In a stringent test of our strategy, we co-cultured HF cells with an excess of MTAP-negative A549-r cells in the presence of DAP and 5′-dAdo. While HF cells were protected from DAP toxicity, A549-r cells were not, and their viable cell numbers decreased markedly (Figure 2B). Similarly, co-cultures of another MTAP-negative cell line, MCF-7, with HF cells, in the presence of DAP and MTA, killed the MCF-7 cells and gave rise to apparently pure cultures of HF cells (Figures 3,4).

We also show that adenine produced by the MTAP substrate 5′-dAdo protected MTAP-positive cells from the toxic effects of 5-FU or 6-TG (Figure 5a,b,c,f). For 5-FU, we show this protection for two different types of MTAP-positive cells: ML-1 and HF (Figure 5a,b). The results with 5-FU and 6-TG are consistent with the interpretation that the abundant adenine supplied by 5′-dAdo competes with 5-FU or 6-TG for PRPP, preventing conversion of these analogs to their toxic products. In addition, 5′-dAdo protected HF cells not only from the toxicity of 6-TG alone, but also from the combination of 6-TG with DAP (Figure 5c,f). Application of such combinations may provide not only synergistic tumor killing but may also decrease the risk of resistance emerging during treatment.

6-TG, 5-FU, and prodrugs, such as capecitabine, have, of course, a long history of clinical applications. A trial of our strategy to enhance the effective treatment of MTAP-negative tumors, by 6-TG or 5-FU, would require a clinical test of just one new type of drug: MTA, or some other MTAP substrate.

### Is MTAP active in serum?

If MTAP activity in serum were high enough to convert administered MTA (or another MTAP substrate) to significant levels of adenine, this might indiscriminately protect not just MTAP-positive host cells, but MTAP-negative tumor cells as well. MTAP was reported to be present in human serum [26]. But MTAP has an absolute requirement for phosphate, and at physiological levels of phosphate, MTAP activity in human serum was found to be negligible [27].

### Toxicity of MTA *in vivo*

MTA has been tested in mice and rats and found to be non-toxic at high doses even when given over extended periods [28], [29]. MTA has also been administered orally to ten humans at 1,600 mg/kg daily, for one month, and seems to be nontoxic [30]. In mice, after intraperitoneal administration at 75 mg/kg, serum levels of MTA reached a peak of 28 µM rapidly and, at 30 minutes, MTA was still at 10 µM [29]. These levels are comparable to those shown to protect HF cells in culture from toxicity of adenine analogs and 6-TG (Figures 2A, 5). After intraperitoneal doses of 75 mg/kg daily, for 28 days, no toxicity was found [29].

MTA is a potent inhibitor of polyamine biosynthesis and produces dose-dependent inhibition of cell growth in culture [31]–[33]. As noted above, it is not toxic to mice, at least at repeated bolus doses of 75 mg/kg [29]. We found that it was not toxic at 50 to100 mg/kg and that it protected mice from lethal doses of 6-TG [34].

The strategy presented here does not depend entirely on use of MTA. More than three dozen MTA analogs have been synthesized [35]–[39]. There may be many that are good candidates for testing.

### Optimization of therapy

In mouse studies by others, F-Ade, MeP, and MTA, when given intraperitoneally, were rapidly taken up by tissues [22], [29]. We found that treatment for four hours with either F-Ade or MeP was highly cytotoxic (data not shown). For such toxic analogs, that are absorbed and act rapidly, our proposed strategy may be most effective if protective MTA (or another MTAP substrate) is given as a bolus dose, followed at some interval by a bolus dose of the toxic analog. Further improvements may be made by using combinations of analogs.

Inhibitors of purine biosynthesis, such as methotrexate, are known to increase the effectiveness of several chemotherapeutic agents by raising PRPP levels [40]. Pretreatments with methotrexate may reduce the amount of analog needed for therapy and also reduce the amount of MTAP substrate needed to protect the host. Our strategy is directed at treatment of MTAP-negative tumors. There are potent inhibitors of MTAP which, if targeted to MTAP-positive tumor cells, could effectively make them phenotypically negative and susceptible to our treatment [41].

We have studied just a few toxic purine and pyrimidine analogs, but many others are known or may be synthesized. Those that deserve further study, because of their demonstrated anti-tumor activity, include 6-mercaptopurine, 8-azaguanine, and 8-azaadenine. Of the many known MTAP substrates, we have tested just MTA and 5′-dAdo. The substrate 9-beta-D-erythrofuranosyladenine has only slight toxicity to hematopoietic progenitor cells in human bone marrow, and is a promising candidate for use with our strategy, *in vivo* [42].

## Materials and Methods

### Cell sources and culture

Colleagues at Dartmouth Medical School generously supplied cells: MTAP-positive human dermal fibroblasts (HF), and the myeloblastic leukemia cell line (ML-1) and MTAP-negative non-small cell lung carcinoma (A549) and breast carcinoma (MCF-7). A ouabain-resistant line, A549-r, was made in our laboratory by n-methyl-n′-nitro-n-nitrosoguanidine mutagenesis and selection in ouabain. This cell line grows well in 0.2 µM ouabain.

For adherent HF, A549, A549-r and MCF-7, cells were cultured in 6 cm-diameter tissue culture dishes at 37°C in a humidified 5% CO_2_ atmosphere, in Dulbecco's Modified Eagle's Medium (DMEM) with 10% fetal bovine serum (FBS, dialyzed against 20 volumes of isotonic saline, with four changes over two days), penicillin (50 units/ml) and streptomycin (50 µg/ml). For experiments with adherent cell lines, cells were seeded into dishes and, one day later, by which time the cells had attached to the substrate, medium was replaced with 4 ml of fresh medium and drugs added as given in the Legends. ML-1 cells were seeded from stocks and grown in suspension in RPMI-1640 with 10% dialyzed FBS. Media, chemicals, and drugs were obtained from Sigma-Aldrich and 2-fluoroadenine from the Drug Synthesis and Chemistry Branch/ Developmental Therapeutics Program/Division of Cancer Treatment, National Cancer Institute.

### Cell growth and viability

At the end of an experiment with adherent HF, A549, and A549-r cells, culture dishes were trypsinized for determination, with a Coulter counter, of cell numbers, or diluted serially into complete medium for clonogenic assay. After a period sufficient for clones to reach a size visible by eye (two to three weeks), they were stained with a solution of crystal violet in saline/methanol. From these counts and from the dilution factors, the numbers of viable cells in the parent cultures were calculated. For photomicroscopy, culture dishes were rinsed with saline and fixed by the addition of 5% glutaraldehyde/saline.
