# Correction: Selective Killing of Tumors Deficient in Methylthioadenosine Phosphorylase: A Novel Strategy

**DOI:** 10.1371/annotation/54fad81d-c975-4b30-bb2b-249650ec3d66

**Published:** 2010-04-28

**Authors:** Martin Lubin, Adam Lubin

This sentence (in Discussion section, "Toxicity of MTA in vivo" subsection) contains an incorrect unit and should appear as follows: "MTA has also been administered orally to ten humans at 1,600 mg daily, for one month, and seems to be nontoxic [30]."

